# Cynomolgus Monkey (*Macaca fascicularis*) Embryo Models: From Developmental Benchmarks to Functionally Testable Embryoids

**DOI:** 10.1111/jmp.70123

**Published:** 2026-10-25

**Authors:** Yeojeong Jeong, Jongwon Byun, Sung‐Hwan Moon, Taeho Kwon

**Affiliations:** ^1^ Department of Animal Science and Technology Chung‐Ang University Anseong Republic of Korea; ^2^ Quantum AI Bio Research Laboratory (KJQI‐JQL), Futuristic Animal Resource and Research Center Korea Research Institute of Bioscience and Biotechnology (KRIBB) Cheongju Republic of Korea; ^3^ Advanced Bioconvergence Department KRIBB School, University of Science and Technology (UST) Daejeon Republic of Korea

**Keywords:** cynomolgus monkey, developmental fidelity, non‐human primate, spatial transcriptomics, stem cell‐based embryo model

## Abstract

Stem cell‐based embryo models now reconstruct increasingly advanced stages of mammalian development, but developmental fidelity remains a central limitation. This review examines why cynomolgus monkey systems are biologically necessary, how natural embryos and emerging technologies benchmark primate development, and how advanced monkey embryoids can strengthen interpretation of human embryo models.

## 1 Introduction

Stem cell‐based embryo models (SCBEMs) have rapidly expanded the experimental study of mammalian development. Mouse models can reproduce post‐gastrulation morphogenesis, including features of neurulation and early organogenesis [1, 2]. Human SCBEMs have similarly progressed from blastocyst‐like structures to implantation and post‐implantation models [3, 4]. Advanced human systems also contain embryonic and extraembryonic compartments [5, 6].

However, model complexity has advanced faster than biological validation. Non‐human primates (NHPs), particularly cynomolgus monkeys ( *Macaca fascicularis* ), address this gap for biological as well as practical reasons. Primate development differs from mouse development in pluripotent‐state progression and in the organisation of post‐implantation embryonic and extraembryonic tissues [7, 8]. After implantation, mouse embryos adopt an egg‐cylinder architecture, whereas monkey and human embryos retain a flatter embryonic‐disc organisation, creating different spatial relationships among the epiblast, amnion, yolk sac, and trophoblast. These architectural differences influence gastrulation, axial patterning and early organogenesis; primordial germ‐cell specification is associated with the nascent amnion, and amnion‐derived signals contribute to primate mesoderm formation [9, 10]. Cynomolgus systems therefore combine natural, stage‐matched primate benchmarks with experimentally accessible stem cell‐derived models. Their value lies in testing which developmental features are conserved, primate‐associated or model‐dependent rather than simply occupying an intermediate position between mouse and human.

## 2 Natural Primate Embryos and Current Technologies

The usefulness of monkey SCBEMs depends on natural‐embryo references used to evaluate them. Long‐term in vitro culture of cynomolgus embryos extended direct observation beyond implantation and into gastrulation [11, 12]. However, limited embryo availability and restricted experimental manipulation constrain the questions that can be addressed using natural embryos alone. Single‐cell RNA sequencing across cynomolgus gastrulation and early organogenesis identified major germ‐layer derivatives, extraembryonic populations and developmental trajectories, while enabling cross‐species comparison with mouse and human datasets [13]. Spatial transcriptomics added positional information that is lost after tissue dissociation. In marmoset gastrulation, spatial profiling resolved regionalised signalling and axis‐associated programmes in utero [14]. Three‐dimensional culture of natural cynomolgus blastocysts later supported neurulation and early organogenesis‐related development, linking molecular identity to tissue‐level morphogenesis [15]. More recently, a three‐dimensional spatial transcriptome atlas of cynomolgus Carnegie stages 9 and 10 mapped early organogenesis in situ and enabled comparison of conserved and species‐specific programmes [16]. In contrast, direct genetic lineage tracing, continuous live imaging and embryo‐scale multiomic profiling remain comparatively limited at advanced NHP stages, whereas existing single‐cell and spatial studies have resolved developmental trajectories, positional signalling and tissue organisation. The priority is therefore not simply to introduce new technologies, but to combine existing molecular and spatial benchmarks with dynamic lineage information so that developmental trajectories, cell–cell interactions and morphogenesis can be tested directly rather than inferred from transcriptomic similarity alone.

## 3 Advancements in Monkey Stem Cell‐Based Embryo Models

Cynomolgus blastoids generated from naïve pluripotent stem cells reproduced major blastocyst lineages and displayed peri‐implantation and gastrulation‐associated potential [17]. Monkey SCBEMs have progressed from blastocyst‐like structures towards models with longer developmental duration, better reproducibility and functional testing [18]. A blastoid‐capsule platform using reprogrammed monkey pluripotent stem cells increased formation efficiency and introduced microfluidic production, illustrating a shift from proof‐of‐concept structures towards more reproducible model generation [19].

A major biological limitation nevertheless remained: robust primate SCBEMs rarely progressed beyond early gastrulation, restricting access to axial organisation, neurulation‐related events and emerging organ progenitors. The recent late‐gastrulation cynomolgus embryoid system directly addressed this limitation. Stem cell‐derived monkey blastoids were maintained to day 25 and developed features broadly corresponding to Carnegie stages 8–9 [20]. Single‐cell analysis showed broad correspondence between embryoid lineages and natural monkey embryos. Perturbation of *EOMES* and *TBXT* also affected blastoid formation, trophoblast differentiation and later mesodermal or endodermal programmes.

The day‐25 embryoids did not contain a distinct node population; trophoblast representation declined during prolonged culture, and development was not sustained beyond day 25 [20]. In parallel, cynomolgus totipotent blastomere‐like stem cells have been used to generate organogenetic embryoids with neural‐tube‐like organisation and cardiac‐like activity, extending the accessible window towards early organogenesis [21]. The field therefore spans blastoids to organogenesis‐oriented systems, although no single model reproduces the complete developmental programme.

## 4 Developmental Fidelity and Relevance to Human Embryo Models

As monkey SCBEMs become more advanced, developmental fidelity should be assessed as a multidimensional property. Expected lineages or transcriptomic similarity alone are insufficient. Evaluation should consider lineage composition, spatial organisation, developmental timing, signalling interactions, embryonic‐extraembryonic coordination, epigenetic state and functional response to perturbation (Figure 1). Existing standards similarly emphasise combined molecular, structural and functional assessment [22]. Current monkey SCBEMs are characterised mainly by lineage‐resolved transcriptomics and emerging functional perturbation; epigenetic equivalence, sustained spatial patterning, signalling interactions and embryonic‐extraembryonic coordination remain less comprehensively validated. Models should therefore be compared with stage‐matched natural embryos rather than judged mainly by culture duration or morphological complexity.

**FIGURE 1:**
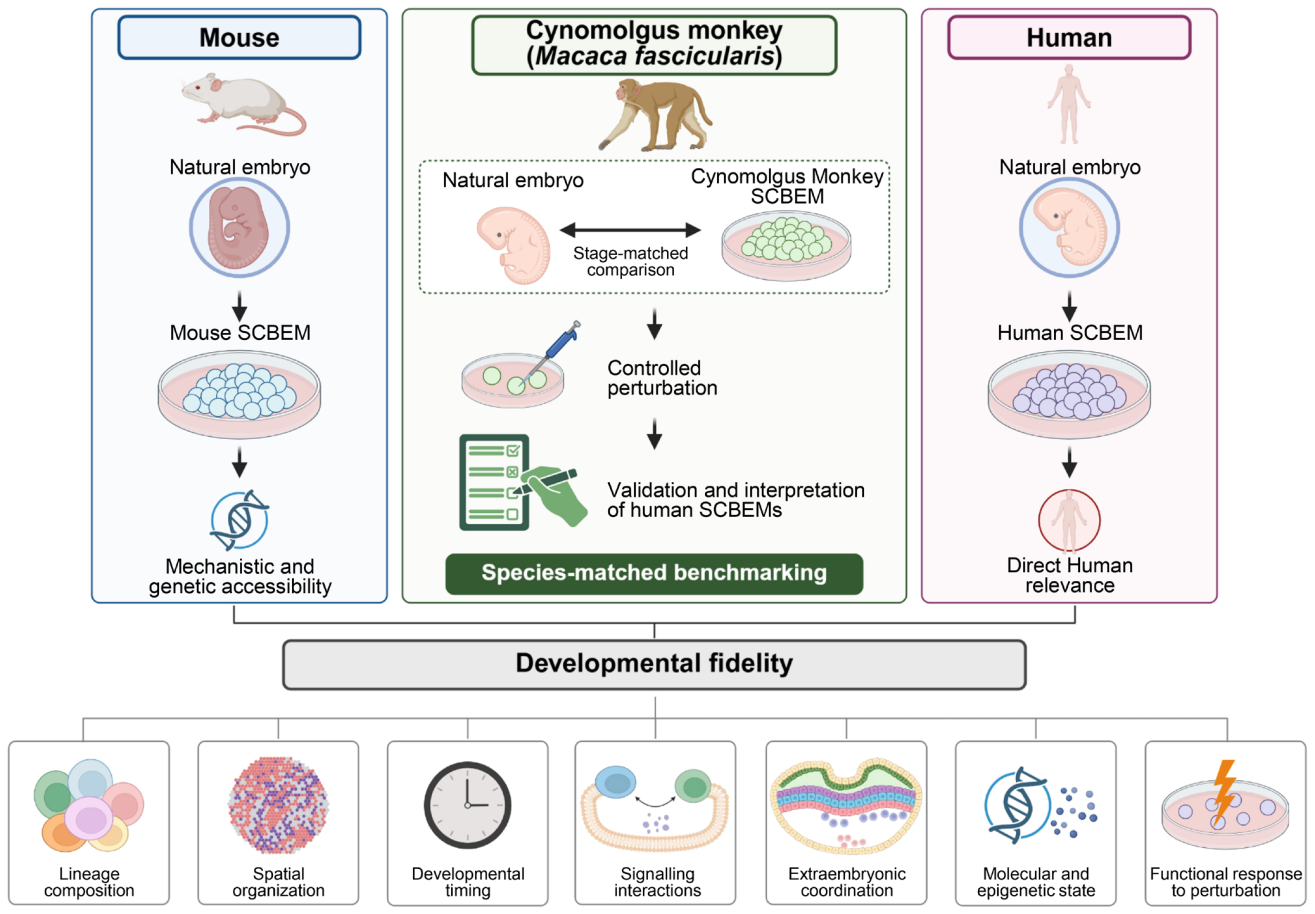
Species‐matched benchmarking of cynomolgus monkey embryo models for multidimensional assessment of developmental fidelity. Mouse, cynomolgus monkey ( *Macaca fascicularis* ) and human embryo systems provide complementary platforms for studying mammalian development. Mouse models offer extensive mechanistic and genetic accessibility, whereas human stem cell‐based embryo models (SCBEMs) provide direct human relevance. Cynomolgus systems enable stage‐matched comparison between natural embryos and experimentally perturbed monkey SCBEMs, providing a primate‐specific benchmark for validating and interpreting human embryo models. Developmental fidelity should be evaluated across lineage composition, spatial organisation, developmental timing, signalling interactions, embryonic‐extraembryonic coordination, molecular and epigenetic state, and functional response to perturbation.

This framework clarifies the distinct roles of monkey and human embryo models. Human SCBEMs provide direct access to human genetic and cellular programmes and are experimentally manipulable, but their validation against stage‐matched natural embryos becomes increasingly limited at later developmental stages [23]. Cynomolgus systems permit species‐matched comparison between natural embryos and experimentally perturbed SCBEMs before findings are compared with human models. These systems are therefore complementary rather than hierarchical. Such comparisons are particularly useful for questions involving amnion function, primordial germ‐cell origin, axial organisation and early organogenesis. Features observed only in vitro should remain provisional until compared with natural development.

Future work should prioritise validation quality alongside extension of developmental stage. Longitudinal imaging, lineage tracing, spatial multiomics and improved maintenance of trophoblast and other extraembryonic compartments are needed to determine whether longer‐lived embryoids preserve normal developmental trajectories. Natural NHP material will remain necessary for stage‐matched benchmarking, while reproducible SCBEMs may reduce repeated embryo use and enable genetic or pharmacological perturbation consistent with replacement, reduction and refinement [24].

## 5 Conclusions and Future Directions

Embryo models now reproduce increasingly advanced gastrulation and organogenesis‐related events, making developmental fidelity a central challenge. Cynomolgus systems address this problem because natural embryos, molecular atlases and experimentally accessible SCBEMs can be compared within the same primate species. NHP embryo models should therefore be viewed not as intermediate substitutes, but as complementary platforms for validating and interpreting primate and human development.

## Funding

This research was supported by the KRIBB Research Initiative Program, funded by the Korea Research Institute of Bioscience and Biotechnology (KRIBB) (KQM0042611 and KQS0092611); the National Research Foundation of Korea (NRF), funded by the Korean government (MSIT), through grant RS‐2021‐NR057659 and the Bio & Medical Technology Development Program (RS‐2023‐00220207); and the National Research Council of Science & Technology (NST), funded by the Korean government (MSIT) (GTL24023‐000).

## Ethics Statement

Ethics approval was not required for this review because it did not involve new experiments using human participants, animals or embryos.

## Conflicts of Interest

The authors declare no conflicts of interest.

## Data Availability

Data sharing is not applicable to this article, as no new data were created or analysed in this review.
